# Integrated Diabetes Care Delivered by Patients – A Case Study from Bulgaria

**DOI:** 10.5334/ijic.2475

**Published:** 2017-03-31

**Authors:** Verena Struckmann, Francesco Barbabella, Antoniya Dimova, Ewout van Ginneken

**Affiliations:** 1 Berlin University of Technology, GE; 2 Department of Health Care Management, Secretariat H80, Straße des 17. Juni 135, 10623 Berlin, GE; 3 Department of Health and Caring Sciences, Linnaeus University, SE; 4 National Institute on Health and Science on Ageing (INRCA), IT; 5 Varna Medical University, BG; 6 Department of Health Economics and Management, Medical University of Varna, 55 Marin Drinov St., 9002 Varna, BG; 7 WHO Observatory on Health Systems and Policies, Berlin University of Technology, GE

**Keywords:** Integrated diabetes care, multiple chronic diseases, health and social care, volunteers, coordination of care, free care

## Abstract

**Introduction:**

Increasing numbers of persons are living with multiple chronic diseases and unmet medical needs in Bulgaria. The Bulgarian ‘Diabetic care’ non-profit (DCNPO) programme aims to provide comprehensive integrated care focusing on people with diabetes and their co-morbidities.

**Methods:**

The DCNPO programme was selected as one of eight ‘high potential’ programmes in the Innovating Care for People with Multiple Chronic Conditions (ICARE4EU) project, covering 31 European countries. Data was first gathered with a questionnaire after which semi-structured interviews with project staff and participants were conducted during a site visit.

**Results:**

The programme trains diabetic patients to act as carers, case managers, self-management trainers and health system navigators for diabetic patients and their family. The programme improved care coordination and patient-centered care by offering free care delivered by a multidisciplinary team. It facilitates the collaboration between patients, volunteers, health providers and the community. Internal evaluations demonstrate reduced hospital admissions and avoidable amputations, with consequent cost savings for the health care system.

**Conclusion:**

Integrated care provided by volunteering patients can empower people suffering from diabetes and their co-morbidities and address health and social inequalities in resource-poor settings. It can also contribute to an increased trust and improved satisfaction among vulnerable patients with complex care needs.

## Introduction

Several studies have observed a rising prevalence and burden of multimorbidity in Europe, although estimates vary across countries [1,2,3,4,5,6]. A recent study has shown a higher prevalence of multiple chronic conditions in Eastern European compared to Western European countries [7]. In Bulgaria, which has one of the lowest life expectancies at birth in the WHO European region with 75 years in 2013 [8], non-communicable diseases impose a large health burden. Cardiovascular diseases (CVD) are attributable for almost 70% of the mortality rates [9]. Probable causes for the increase of non-communicable diseases are recent changes in life style, such as increasing tobacco, alcohol and drug consumption, low physical activity, as well as socioeconomic inequalities in accessing appropriate health care services [10]. The rise of persons with chronic diseases and multimorbidity requires better coordination of care between various health and social care providers and sectors. Yet the Bulgarian health system has long been plagued by care fragmentation, underfunding of health care services, low quality of care and large socioeconomic inequity in access [10]. These problems increase the need for innovative solutions, in particular for vulnerable population groups. Although the government has recently rolled out the National Health Strategy for Prevention of Chronic Non-Communicable Diseases 2014–2020 [11], no specific policies have been implemented at national or regional level until now.

Against this background, in 1994, a programme named ‘Diabetes has no limits’ was started in Burgas – the fourth largest Bulgarian city with a growing population of about 200,000 inhabitants. It was the initiative of a nurse, a cardiologist, a general practitioner (GP), an ophthalmologist and four diabetic patients. Since then, the name was changed into the Bulgarian ‘Diabetic care’ non-profit programme (DCNPO) (PCHЦ “Диабетни грижи” – Бургас) and the range of services provided was gradually expanded. The DCNPO targets diabetic patients with co-morbidities and their families, but also the general population [12]. The DCNPO is the only programme that offers inexpensive, easy-to-access integrated care for individuals living with diabetes and their co-morbidities in the Burgas region. One of the unique features is the way it trains diabetes patients to become case managers, self-management trainers and health system navigators. The programme’s stated goals are to enhance the quality of life of diabetic patients, to increase their ability to cope independently with diabetes and relevant co-morbidities and to assist their integration into society [12]. This article aims to provide an in depth description of the innovative features of the Bulgarian DCNPO programme and concludes with lessons to be learned.

## Methods

For this case study we used data from the European ICARE4EU (Innovating care for people with multiple chronic conditions) project. This project was initiated in 2013 to contribute to the innovation of care for European citizens with multiple chronic conditions by gaining more insight into potentially effective and efficient patient-centered, multi-disciplinary care approaches that have been developed and implemented in Europe.

Expert organizations in 31 European countries identified integrated care programmes that provided care for people with multimorbidity. Inclusion criteria for these programmes were:

1. Target adult people with multimorbidity, defined as two or more medically (i.e. somatic and/or psychiatric) diagnosed chronic (not fully curable) or long lasting (at least six months) diseases, of which at least one of a (primarily) somatic nature;
2. Include formalized collaboration(s) between at least two services, including medical services;
3. Evaluated or planned to be evaluable in some way;
4. Currently running or finished less than 24 months ago or starting within the next 12 months.

According to the above mentioned selection criteria, the ICARE4EU project identified 112 eligible integrated care programmes for patients with multimorbidity in 25 European countries (out of 31 countries). Information on programmes was collected with the support of expert organizations/programme managers in each country included in the study. They were asked to search and report all integrated care programmes focusing on multimorbidity in their country. An online questionnaire was available in eleven languages and contained questions on several programme characteristics (e.g. general information, patients, quality and evaluation), including aspects related to eHealth tools eventually adopted within the programmes themselves. In a second step, promising practices were selected for a site visit and further study. To this end, the project team scored the programmes on five dimensions: (1) a general score (e.g. evaluation design, perceived sustainability and transferability), and an indication of its level of (2) patient-centeredness, (3) integration of care, (4) use of eHealth technologies and (5) its innovativeness in financing mechanisms. This resulted in a short list of eight ‘high potential’ programmes to be visited, among them the Bulgarian DCNPO programme.

Our case study was conducted in May 2015 by means of semi-structured interviews, and a site visit to the DCNPO in Burgas. We held interviews with a range of stakeholders involved in the DCNPO programme, including the manager, the deputy chairperson, a specialist, care managers, and volunteers. A total of seven staff members were interviewed. The semi-structured interviews lasted between 40 and 90 minutes. They were conducted in English by two of the authors (VS and FB) and simultaneously translated by another author who is a native Bulgarian speaker (AD). They were digitally recorded and transcribed by two different persons. All names were replaced with numbers in order to guarantee anonymity. The participants had the right not to answer and could stop the interview at any time.

## Description of the care practice

### Programme background and target group

The Bulgarian DCNPO was founded to respond to the need of chronic diabetes patients with co-morbidity for accessible, coordinated and comprehensive medical and social care. The DCNPO approach consists of building a community of diabetes patients and health service providers, who are self-empowered and capable of dealing with complex patients’ needs. This approach differs from most Bulgarian non-governmental (NGOs) or non-profit organisations (NPOs), which usually advocate the rights of certain vulnerable patient groups and heavily rely on organizational and financial support from institutions. Indeed, the DCNPO takes a more active role and trains and educates patients in self-management of their chronic condition. Many of them also work as volunteers and provide social and health care assistance (e.g. diabetes management, arrange appointments), help patients navigate the health system and train informal carers. Through the involvement of patients, family members and specialists, the DCNPO achieves strong collaborations and synergies, which allow building on voluntarism rather than the government. Other important elements that characterize the programme are a focus on prevention, care integration, shared decision making and patient-centeredness [12].

Although the programme primarily targets individuals with diabetes, many of the patients served by the DCNPO have multiple chronic diseases (e.g. heart disease, cancer, depression). The DCNPO therefore has increased its scope of services and aims to provide comprehensive and coordinated care to the whole population. Generally, people come from lower socio-economic groups and many have no health insurance [12].

### Care delivery and scope of services

As of 2016, the DCNPO serves 1,600 people, of whom 416 are “active” members in the sense that they pay small (€5) contributions and participate in the social activities organized by the DCNPO, such as meetings, trips and conferences. As of 2016, there are 15 volunteers and three paid care managers, providing the core of the DCNPO’s services, including diabetes and additional chronic disease related acute and long term care services. The programme is directed by a retired former nurse who also chairs the 7 person board, which is made up of DCNPO volunteers. The three care managers permanently staff the DCNPO site and form the first point of contact for new patients. They carry out comprehensive physical examinations and assess the care needs and preferences of the patients. After this first assessment, the volunteers become the patient’s focal point. They provide treatment, make home visits, coordinate services and arrange appointments for specialised treatment. After each home visit, the responsible volunteer informs participating care professionals and shares relevant information for a continuous care process. The volunteers also provide training and support to empower patients in becoming active partners in their care process but also in eventually becoming volunteers. Volunteers and care managers are always available for the patients since they can visit and call the DCNPO the entire week. In addition, over 20 care professionals (GPs, specialists, nurses, psychologists, etc.) collaborate with the DCNPO, on a voluntary basis or where possible under health insurance. Together with the professionals, the DCNPO provides a wide spectrum of services (see **Box 1**) [12].

**Box 1: Scope of services covered by DCNPO [12]**

– Diabetes mellitus type II management
– Cancer management in collaboration with other NGOs in Burgas
– Lifestyle intervention for patients with diabetes and cardiovascular diseases
– Education and training of informal carers
– Provision of social care at home
– Home and office based health services
– Diabetic foot management and care
– Active health promotion for patients
– Health education for students and the elderly
– Psychotherapeutic/psychiatric care
– Coronary heart disease management in collaboration with the Cardio Centre Pontika (volunteering cardiologist)
– High blood sugar screening programmes for the general population
– Promotion of patient self-management abilities

Currently, the DCNPO is increasingly focusing on childhood diabetes and has started preparing promotional diabetes campaigns in schools with a focus on nutrition, physical activity, obesity and overweight. Furthermore, both volunteers and patients participate in further developing and assessing the programme [12]. To improve its services, the DCNPO is a founding member of the ‘blue circle of hope’ (see **Box 2**) and cooperates with several other diabetic care programmes operating in other areas of Bulgaria or abroad [12,13].

**Box 2: Blue circle of hope**

The “Blue circle of hope” is a network of various diabetic care programmes and organizations from different countries and regions, which was started by the DCNPO in 2011. Its name is inspired by the symbol for diabetes (a blue circle), initially developed for a United Nations campaign. The network started off with ten Bulgarian organisations but has been gradually expanding and now has members from Italy, Russia, Serbia, Turkey, and Ukraine. The network schedules regular meetings where daily experiences are shared and latest evidence about diabetes, its prevention and treatment options are discussed. Lastly, the DCNPO invests a great deal of effort in the development of the network and ultimately hopes to establish it as a regional centre for diabetic care that can perform projects and research.

### Training of volunteers and care managers

Care managers and volunteers basically receive the same training. Specialized training is provided by physicians to younger diabetes patients who could also offer care to others. Their training is comprehensive and consists of continuous individual and group training. The training programmes are based on continuing education programmes by the World Health Organization (WHO) [14] and cover care for patients with diabetes and other chronic problems (e.g. cardiovascular diseases), patient self-management and shared decision-making. In addition, conferences are organized where latest evidence is shared.

Since 1997, physicians who cooperate with the DCNPO have trained 47 unemployed diabetic patients, to act as care managers. They are employed as assistants on a temporary labour contract basis under a programme governed by the Ministry of Labour and Social Policy. This programme aims to ensure employment of people with disabilities or chronic diseases. Currently, three care managers are working for the DCNPO under this programme. In addition, over the same time period, 25 volunteers have been trained to act as volunteers in the programme. Currently, 15 of them are active. Although they receive a similar training, the latter make the home visits and do the follow-ups. They do not work as care managers at the DCNPO site because they have jobs of their own and are only part time volunteers who therefore require flexible hours.

### Care integration and patient-centeredness

Establishing a provider network capable of providing seamless comprehensive and patient-centered care has taken several years. For many patients in need of care, the DCNPO is the first entry point to the health system. As de facto gatekeeper, the DCNPO can help avoid unnecessary interventions and improve care coordination and continuity for patients. In addition, the DCNPO has enhanced patient-centeredness through the involvement of patients, family and informal carers in the care and decision-making process. This should ensure effective collaborations and secure their commitment to the care plan [12]. Furthermore, regular communication with community groups, associations, self-help groups, and educational centres allows a continuous holistic assessment of the patient and care approach.

### Financing of the programme

Funding of the DCNPO is quite unconventional. First, the DCNPO receives an annual subsidy from the municipality of Burgas. Funding from the municipality has to be applied for and as a result may vary in size. It has been approximately €2,000 per year the last couple of years [12]. This subsidy is independent of the DCNPO’s performance, health outcomes or any achieved savings. Second, DCNPO members pay annual contributions of €5 per person. This constitutes the only stable revenue source. Third, the DCNPO receives funds from the MLSP for the salaries of the three care managers. Lastly, the DCNPO also receives private donations. However, these have decreased in recent years as a result of the financial crisis and a legislative change that limited deducting donations to charity from tax. This implies that the raised revenue is inadequate to cover all activities and most work is therefore performed on a voluntary basis [12].

### Impact

Until now the impact of the programme has only been evaluated internally and therefore the following outcomes have to be interpreted with caution. The model with diabetic patients acting as volunteers, shared responsibility between patient and professional and continuous learning has altered the patient-provider relationship. Contrary to the often quite hierarchical and traditional regular care system in Bulgaria, the DCNPO model has led to increased trust and patient satisfaction [12]. DCNPO patients adhered better to treatment and had better health outcomes than patients treated in the regular system. The collaborative approach involving the patient, their relatives and informal carers enables better monitoring and follow-up of constantly changing patient needs. Moreover, since 2010 alone, DCNPO activities seemed to have helped preventing about 400 amputations and thus avoidable hospital admissions in complex diabetes cases [12]. This estimation is based on photo documents, patients’ dossiers and professional assessments. The cost savings for the health system, based on the number of prevented amputations and the tariff paid by the National Health Insurance Fund (NHIF) per procedure, were calculated at about €200,000. Another positive outcome is that the programme has decreased inequities because vulnerable groups are targeted, some without health insurance, and care is offered free at the point of delivery. These estimations sound credible, as a programme developed by Stanford University (USA), which has some similarity in that it uses chronic patients to train other chronic patients in self-management, has shown similar improvements [15,16].

## Discussion

The Chronic Care Model (CCM) proposed by Wagner et al. (1996), perhaps the most used model for chronic care programmes, suggests that such programmes ideally comprise six interlinked key elements [17,18]. The DCNPO programme contains many of these elements. Indeed, the first element, self-management support, is reflected in the involvement of patients in decisions and the design of their care plan, but also in the provision of health education and self-management training to patients and their families. The second element, delivery system design, broadly focuses on clarifying roles and tasks of all involved, making sure that all the physicians have centralized and up to date information, and that follow-ups are an integral part of the care process. This element is visible in the way care managers and volunteers act as case managers who work in multidisciplinary teams and schedule regular follow-ups and home visits. Although a continuous sharing of relevant information, e.g. after follow-ups and visits, is a key part of the care process, this is not supported by a centralized system. The third element, decision support, is implemented by means of treatment guidelines for providers and training programmes for care managers and volunteers, which are based on WHO guidelines. The fourth element, a clinical information system, does not exist as the DCNPO is not yet using any form of electronic communication technology in their daily work. Until now, information exchange takes place using written notes, telephone, or face-to-face conversations. Moreover, patient records are still in paper form and not electronic. The DCNPO could benefit from a better information infrastructure and low-cost mobile health technology but these remain unaffordable. The fifth element, the health system, focuses on ensuring that senior management, staff and volunteers all visibly support and promote the improvement of chronic care. This element can be viewed as a hallmark of the DCNPO. Without strong leadership, the programme could not have operated in an environment that lacks financial support and where many health professionals still remain hesitant to collaborate (also see below for more elaboration). In addition, the initiation of the blue circle of hope can be seen as an initiative promoting the DCNPO’s strong commitment to improving diabetic care. The final element, which relates to effectively using existing community resources, is reflected in several initiatives by the DCNPO to involve the local community, e.g. by offering prevention programmes in schools. Using Wagner’s CCM model, the DCNPO can thus be characterised as a successful programme as it integrates many of its fundamental elements, although some, most notably those relating to an effective information system, are missing.

The future for the DCNPO is not without challenges. Firstly, the DCNPO cannot perform effective medium- or long-term financial planning because municipal funding and private donations fluctuate annually. Therefore its potential to save cost should be demonstrated in external evaluations to convince policymakers of this approach [12]. So far this was never carried out due to limited resources, but perhaps also because the DCNPO managers and volunteers have no doubt that their programme is cost-effective. It is also important to note that legislative changes would be necessary to allow the NHIF to contract integrated care providers such as the DCNPO. Secondly, although the DCNPO is supported by various health professionals, many professionals still seem hesitant to collaborate; perhaps because peer support, health promotion and a self-management approach are not part of their professional culture. A change in culture towards a shared vision is needed if these types of integrated care programmes are to be scaled up nationally [12].

## Conclusion

The DCNPO programme provides integrated care services of good quality to complex patients in a challenging environment with limited financial and human resources. The DCNPO programme suggests that training patient-volunteers to become carers of other patients can contribute to an increased trust in one-another and improved satisfaction and treatment adherence among patients (see **Box 3**). The DCNPO approach has been widely adopted across Bulgaria and various new initiatives have joined the ‘blue circle of hope’ network. This provides strong evidence of the transferability of this model in the region. The DCNPO further demonstrates that networking and coordination of actors and professionals in health care is essential in shifting from disease-centered to patient-centered care. Good working relations, clear roles and shared responsibilities among participating actors and professionals of the DCNPO played an important role in overcoming fragmentation of the health care system. In addition, strong bottom-up leadership is crucial as the DCNPO case evidences. Successful replication relies heavily on the commitment to change and persistence of the local leadership and its members.

**Box 3: Main conclusions**

- Non-profit care provided by volunteering patients can play a major role in addressing health and social inequalities where public health care systems and the socioeconomic context are insufficient
- Trained volunteers who are patients themselves can contribute to an increased trust, respect and improved satisfaction among vulnerable patients with complex care needs
- Successful replication relies heavily on the commitment, and persistence of the leadership and its members but could be especially effective in resource-poor settings

Programmes such as the DCNPO can inspire others to not accept the constraints of resource-poor settings but to realize that improved care for people with diabetes and their co-morbidities can be organized with volunteers and patients. Yet relying on volunteers should not be the goal but rather the means to achieve integration in formal care and thus access to proper funding.

## References

[1] Glynn L, Valderas J, Healy P, Burke E, Newell J, Gillespie P, Murphy A (2011). The prevalence of multimorbidity in primary care and its effect on health care utilization and cost. Family Practice.

[2] Barnett K, Mercer SW, Norbury M, Watt G, Wyke S, Guthrie B (2012). Epidemiology of multimorbidity and implications for health care, research, and medical education: a cross-sectional study. Lancet.

[3] Orueta J, Nuño-Solinís R, García-Alvarez A, Alonso-Morán E (2013). Prevalence of multimorbidity according to the deprivation level among the elderly in the Basque Country. BMC Public Health.

[4] Fortin M, Stewart M, Eve Poitras M, Almirall J, Maddocks H (2012). A systematic review on prevalence studies on multimorbidity: toward a more uniform methodology. Annals of family medicine.

[5] Taylor AW, Price K, Gill TK, Adams R, Pilkington R, Carrangis N, Shi Z, Wilson D (2010). Multimorbidity – not just an older person’s issue. Results from an Australian biomedical study. BMC Public Health.

[6] Mercer SW, Watt GCM (2007). The Inverse Care Law: Clinical Primary Care Encounters in Deprived and Affluent Areas of Scotland. Annals of family medicine.

[7] Afshar S, Roderick P, Kowal P, Dimitrov B, Hill A (2015). Multimorbidity and the inequalities of global ageing: a cross sectional study of 28 countries using the world health survey. BMC Public Health.

[8] World Health Organization Life expectancy at birth 1990–2013. http://apps.who.int/gho/data/node.main.688.

[9] World Health Organization Bulgaria. Country cooperation strategy at a glance. http://www.who.int/countryfocus/cooperation_strategy/ccsbrief_bga_en.pdf.

[10] Dimova A, Rihova M, Moutafova E, Atanasova E, Koeva S, Panteli D, van Ginneken E (2012). Bulgaria Health system review. Health systems in transition.

[11] Ministry of Health http://ncphp.government.bg/en/news-2/868-national-program%20-for-prevention-of-chronic-non-communicable-diseases-2014-2020.html.

[12] ICARE4EU case report Regional non-profit organisation (NPO) “Diabetic care” Burgas, Bulgaria. http://www.icare4eu.org/pdf/Diabetic_Care_Burgas_programme_Case%20Report.pdf.

[13] International Diabetes Federation (2016). Unite for Diabetes. http://www.idf.org/bluecircle.

[14] WHO Regional Office for Europe (1998) Therapeutic Patient Education. Continuing Education Programmes for Health Care Providers in the Field of Prevention of Chronic Diseases, Copenhagen. http://www.euro.who.int/__data/assets/pdf_file/0007/145294/E63674.pdf.

[15] Lorig KR, Ritter P, Stewart AL, Sobel DS, Brown BW, Bandura A (2001). Chronic Disease Self-Management Program: 2-Year Health Status and Health Care Utilization Outcomes. Medical Care.

[16] Lorig KR, Sobel DS, Ritter PL, Laurent D, Hobbs M (2001). Effect of a Self-Management Program on Patients with Chronic Disease. Effective Clinical Practice.

[17] Wagner EH, Austin BT, Von Korff M (1996). Organizing care for patients with chronic illness. Milbank Q.

[18] Wagner EH, Austin BT, Davis C (2001). Improving chronic illness care: translating evidence into action. Health Aff.

